# Unraveling the Role of Immune Checkpoints in Leishmaniasis

**DOI:** 10.3389/fimmu.2021.620144

**Published:** 2021-03-11

**Authors:** Rafael de Freitas e Silva, Esther von Stebut

**Affiliations:** ^1^ Department of Natural Sciences, University of Pernambuco, Garanhuns, Brazil; ^2^ Department of Dermatology, Medical Faculty, University of Cologne, Cologne, Germany

**Keywords:** *Leishmania*, Leishmaniasis, Immune Checkpoints, Co-inhibitory Receptors, Immunotherapeutic

## Abstract

Leishmaniasis are Neglected Tropical Diseases affecting millions of people every year in at least 98 countries and is one of the major unsolved world health issues. *Leishmania* is a parasitic protozoa which are transmitted by infected sandflies and in the host they mainly infect macrophages. Immunity elicited against those parasites is complex and immune checkpoints play a key role regulating its function. T cell receptors and their respective ligands, such as PD-1, CTLA-4, CD200, CD40, OX40, HVEM, LIGHT, 2B4 and TIM-3 have been characterized for their role in regulating adaptive immunity against different pathogens. However, the exact role those receptors perform during *Leishmania* infections remains to be better determined. This article addresses the key role immune checkpoints play during *Leishmania* infections, the limiting factors and translational implications.

## Introduction

Global warming is progressively changing the distribution of pathogenic microbes, and consequently humans are more exposed to new or re-emerging infections, especially those transmitted by vectors and reservoirs (1–3). Leishmaniasis affects millions of people living in endemic regions and kills thousands of them every year, and its distribution is changing due to the occurrence of sandflies in new areas (4–7). Those diseases are listed by the World Health Organization (WHO) as important Neglected Tropical Diseases (NTDs) affecting mainly poor people living with less than one dollar per day (8).

Leishmaniasis are complex parasitic protozoan infections, usually transmitted through the bite of infected hematophagous invertebrates, capable of surviving and proliferating in human tissues and blood (9–11). So far, the best option to control the diseases is through vector and host reservoir control, and by treating infected individuals (12, 13). Nevertheless, the drugs are often inaccessible due to high cost and toxicity and there is currently no safe and effective vaccine to be applied in humans (14–16). One of the reasons for the lack of therapeutic approaches is the complex immunity against these parasites and, therefore, many gaps in understanding this puzzle (17). In this regard, this review aims to address some key pieces which were initially observed in cancer and chronical viral infections.

Development of immunity is accompanied by an upregulation of receptors (and their ligands) with regulatory function, also known as immune checkpoints (18–20). Some of them are potentially stimulatory and others induce an inhibitory effect. For this, it was demonstrated that the upregulation of co-inhibitory receptors and their ligands on antigen presenting cells (APCs) and activated T cells are capable to suppress the immunity by delivering negative signals, rendering T cells suppressed in their function (21–24). Nonetheless, antibody blocking can restore T cell capacity to destroy cancer or viral infected cells. Among those, anti-Programmed Death (PD)-1 and anti-Programmed Death Ligand (PD-L)1 are currently used to treat melanoma and other types of cancer (25, 26). Those treatments paved the way for studies with similar interventions for chronic infections, including leishmaniasis.

This review wants to address the following question: what is the role of immune checkpoints in leishmaniasis? Some investigations are ongoing; however, given the limited understanding of their function, dissecting it would significantly amplify our knowledge regarding the function of the human immune system, how it reacts to the intracellular parasite *Leishmania* and other similar infections. More importantly, it would widen treatment perspectives for the affected individuals.

## An Overview on Anti-Leishmania Immunity

**Figure 1:**
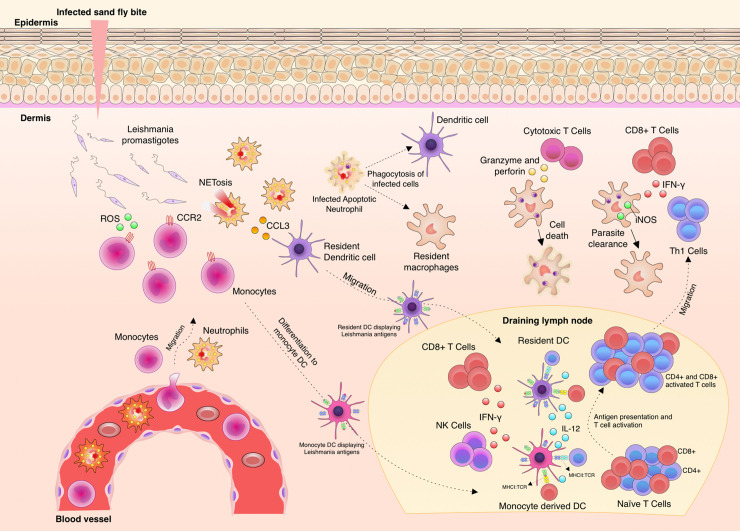
Aspects of immunity against *Leishmania* parasites. Upon *Leishmania* entry into the dermis, different phagocytic cells infiltrate to the site, such as neutrophils and monocytes. The parasites are phagocyted by these infiltrating cells and also by resident macrophages and tissue dendritic cells (DC). Neutrophils produce increasing levels of chemokine (C-C motif) ligand 3 (CCL3) to attract dendritic cells to the site. C-C chemokine receptor type 2 (CCR2)^+^ monocytes produce and release reactive oxygen species (ROS) to kill free parasites. Then, adaptive immunity is elicited through the migration of monocytes and tissue DCs carrying *Leishmania* antigens to the draining lymph node. These cells present parasite antigens and produce Interleukin (IL)-12 and thus induce CD4^+^Th1 cell differentiation, Th1 cells migrate to the infection site and finally produce and secrete Interferon (IFN)-γ. Activation of infected macrophage by the action of IFN-γ leads to the production of nitric oxide (NO) by iNOS and thus *Leishmania* killing. IFN-γ is also locally produced by natural killer (NK) and CD8^+^ T cells. IL-10 production and parasite persistence are necessary to maintain memory cells.

Immunity against *Leishmania* is complex and depends on many factors, such as genetic diversity, parasite species and isolates (27–29). *Leishmania* spp. are inoculated into the skin as metacyclic promastigotes (30) and once the parasites are in close contact with the body, immunity is triggered (**Figure 1**). The complement system has an important, although limited function in this task, since glycoproteins, such as GP63 (also known as Leishmanolysin), from the surface of the parasites are capable to bind complement factor C3b and inactivate it (C3bi), blocking the capacity to lyse the parasites and enhancing its recognition by complement receptor-3 (CR3) on macrophages (31–33). As soon as phagocytic cells reach the entry site, they engulf free parasites and factors such as chemokine (C-C motif) ligand 3 (CCL3) are secreted by neutrophils, which in turn attract dendritic cells (DCs) (34–36). C-C-chemokine receptor type 2 (CCR2)-driven monocytes secrete reactive oxygen species (ROS) to kill free parasites and these cells migrate to draining lymph nodes and differentiate to monocyte-derived DCs (9, 37–39). DCs displaying *Leishmania* antigens coordinate the secretion of interleukin (IL)-12 which instructs the differentiation of T helper type (Th)1 cells to produce and secrete IFN-γ (40–42). IFN-γ levels collectively produced by CD4^+^ Th1 and other activated cells types, such as CD8^+^ T cells and natural killer (NK) cells, is, so far, known as the best correlate of protection in leishmaniasis (43, 44). Protection takes place by production of nitric oxide (NO) by the inducible NO synthase (iNOS) in macrophages in order to kill the amastigotes (45–48).

The role of Th1 cells is well verified in the two main mouse models of *L. major* infection: the susceptible mouse strain BALB/c shows a weak Th1 and strong Th2 immunity that results from the contribution of distinct factors such as an IL-4-mediated down regulation of the IL-12Rβ on Th2 cells or increased production of IL-12(p40)_2_ homodimers that antagonize the effect of the IL-12 active form on IL-12R (41, 42, 49); on the other hand, *Leishmania*-resistant C57BL/6 mice are good controllers of the infection with a strong Th1 response (50–52).

Another important component are Th17 cells and its main cytokine IL-17 that contributes activating iNOS and inducing production of GM-CSF, IL-1β, IL-6, IL-8, TNF-α, and chemokines (53). Th17 cells have been implicated in the recruitment of neutrophils to the lesions caused by *Leishmania*, and disease progression was prevented in IL-17A-deficient BALB/c mice (54, 55). Nevertheless, disease progression was not affected in IL-17-deficient C57BL/6 mice (56).

The role of CD8^+^ T cells is still controversial and has been associated with more inflammation, increased Granzyme B, TNF-α and IL-1β production, inflammasome activation, tissue damage and disease severity (57–59). On the other hand, it has been shown that CD8^+^ T cell-derived IFN-γ is a key factor for disease control (60, 61).

Additionally, regulatory T cells (Treg) are necessary to control the activation of the immune response and excessive inflammation by diverse mechanisms, such as inhibitory receptors and enzymes, and production of IL-10 (62). Moreover, evidence indicates that they are necessary to control the inflammation process and contribute to parasite persistence in resistant mice (63–65). In this regard, it was shown that Langerhans cells (LCs), during *Leishmania* infection, induced expansion of Treg. Moreover, Treg-derived IL-10, retinoic acid independent, contributes parasite persistence and selective depletion of Treg induces larger lesions (66, 67).

In humans, the immunity against *Leishmania* is more complex, and often many findings achieved in the mouse models cannot be directed translated to humans (17, 68, 69). The key players during the immunity in CL and VL are similar, however, the tissue milieu is distinct and that influences the course of immunity and final outcome.

## Main Clinical Manifestations

Even so, in many cases immunity is unable to properly control parasite growth and they end up replicating as amastigotes in macrophage phagolysosomes (70, 71). From the point of inoculation, some species can have a dermis tropism, causing localized or disseminated skin lesions, or mucocutaneous lesions. *L. braziliensis*, *L. major*, *L. panamensis*, *L. mexicana*, and *L. guyanensis* are species associated with these clinical forms. Other Leishmania species have a tropism for the mononuclear phagocyte system from spleen, liver, and bone marrow, and can cause visceral leishmaniasis (VL), which is the deadliest form of leishmaniasis if left untreated (6, 72, 73). Thus different species of the parasite are involved with distinct clinical forms (11, 74).

This broad clinical spectrum adds another layer of complexity to understand immunity against *Leishmania* (28, 75, 76). The activation of the immune system has been used to detect antibodies and/or cellular immunity for diagnostic purposes, and, so far, it is not fully understood how it works to contain the infection and how can we boost it (77–81). Overall, immunity against *Leishmania* parasites is very complex and involves many cellular and molecular players that act against the different species of parasites, which can cause infection, and also the intrinsic factors, that influence it (**Figure 1**).

## What are the Immune Checkpoints in Leishmaniasis?

**Figure 2:**
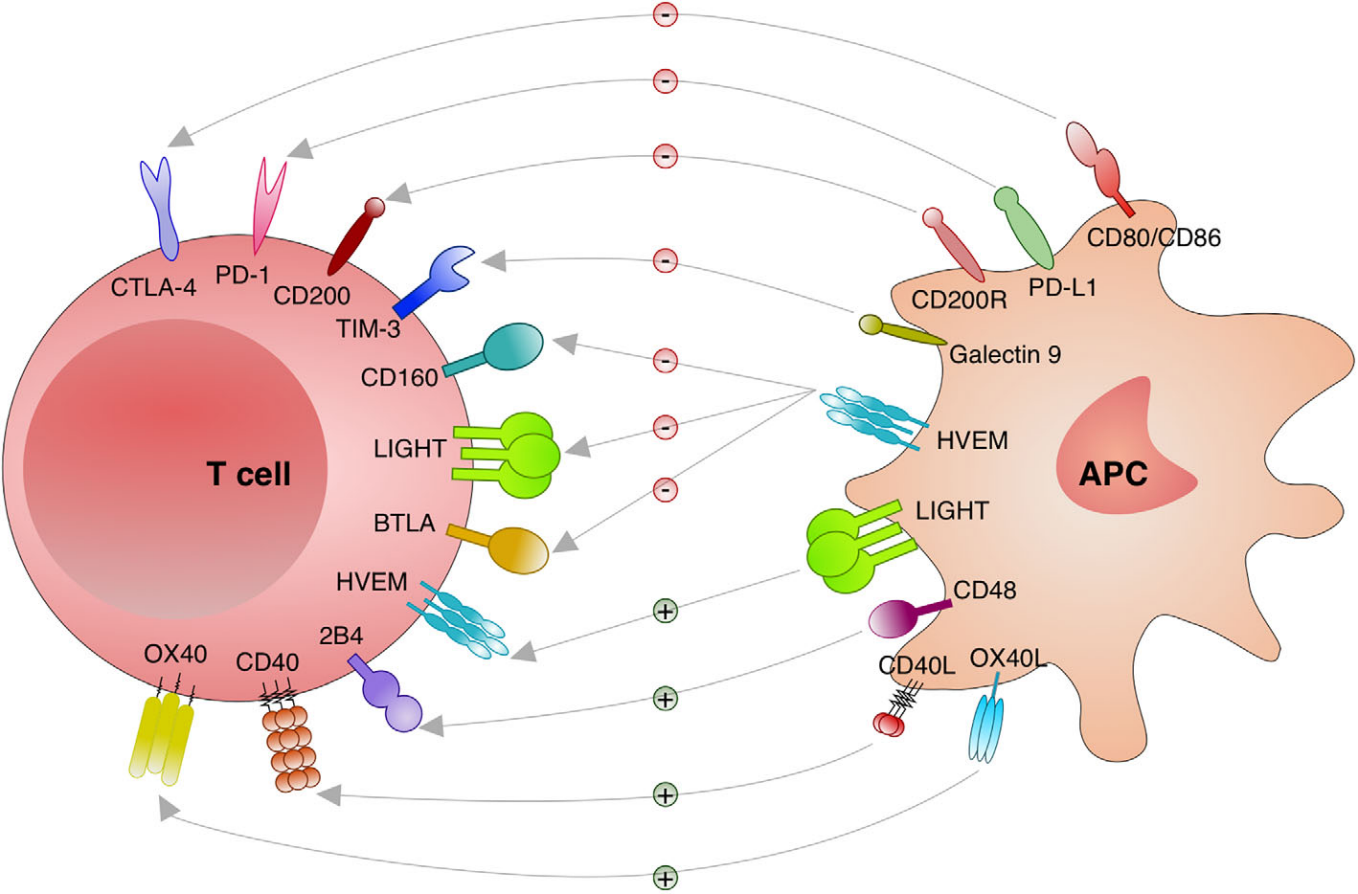
Immune checkpoints investigated in leishmaniasis. Distinct immune checkpoints have been investigated for the function during the immunity against *Leishmania*. Cytotoxic T lymphocyte attenuator 4 (CTLA-4; CD152) binds to CD80/CD86 and blocks its stimulatory effect on T cells. Programmed cell death protein 1 (PD-1; CD279) binds to its ligands PD-L1 or PD-L2 and inhibit T cells. CD200 (OX-2) and CD200R engagement results in an important inhibitory signal to T cells. T cell immunoglobulin and mucin domain-containing protein (TIM)-3 and Galectin 9 engagement deliver an inhibitory signal to T cells. Binding of CD160, lymphotoxin LT-like (LIGHT) and B and T lymphocyte attenuator (BTLA; CD272) to herpesvirus entry mediator (HVEM) induces inhibitory signals in T cells. When HVEM from T cells binds to LIGHT on antigen presenting cells (APC) stimulatory effects on T cells are induced. Engagement of CD40 and CD40 Ligand (L) delivers a stimulatory effect to T cells. 2B4 and CD48 binding results in stimulatory effects to T cells. Binding of OX40 to OX40L results in stimulatory effects to T cells.

Different stimulatory and inhibitory immune checkpoints pathways are activated or deactivated during an immune response. Those interactions are cell to cell (trans), usually between a T cell and a macrophage, DC, monocyte and neutrophil. However, it has been shown that some interactions are on the same cell (cis) with important consequences for the T cells (82). Altogether, different checkpoints receptors have been discovered with distinct functions that are relevant to consider and to investigate in the context of intracellular infections such as *Leishmania* (**Figure 2**).

### Inhibitory Checkpoints

#### Programmed Cell Death 1 (PD-1) and Programmed Death-Ligands 1 (PD-L1) and 2 (PD-L2)

The programmed cell death protein 1 (PD-1; CD279) and its ligands PD-L1 (B7-H1) and PD-L2 (B7-DC), which are members of the B7/CD28 family are important axis which were investigated in leishmaniasis (83–85). Engagement of PD-1 to PD-L1 or PD-L2 induces phosphorylation of tyrosine residues in PD-1 intracellular domain which recruits tyrosine phosphatases such as Src homology 2 domain-containing phosphatase (SHP)-2. Thereafter, distinct kinases necessary for cell survival and proliferation are dephosphorylated (86–89). The resulting effect of this signaling cascade is T cell inhibition and exhaustion (90, 91). It has been shown in different models that PD-1/PD-L1 are important to maintain immune homeostasis by inducing peripheral tolerance and protecting tissues from autoimmune attack (24). The role of these molecules during acquisition of immunity against many infectious agents is not well established and PD-1/PD-L1 may suppress immunity allowing chronic infection (92–94). Infection by *L. mexicana* can cause a localized cutaneous lesion (LCL) or a diffuse form (DCL) and it has been observed that patients with DCL have low numbers of CD8^+^ T cells in their lesions when compared to LCL (83). Moreover, CD8^+^ T cells isolated from the blood of DCL exhibited less capacity to proliferate under antigen specific stimulation, less IFN-γ production, and enhanced expression of PD-1 (83). This cell phenotype observed has been typically associated with CD8^+^ T cell exhaustion (25). However, particularly in this study, other exhaustion markers have not been extensively investigated and this could give a better idea on the level of CD8^+^ T cell exhaustion undergone by those infected patients. During human CL, higher frequencies of CD4^+^PD-1^+^ T cells were observed in active CL disease patients compared to post-treated patients and uninfected individuals (95). Interestingly, it was observed a continuously increasing frequency of PD-1^+^ T cells together with the development of CD4^+^ T cells subpopulations (95). Lower frequencies of PD-1^+^ among T naïve (CD45RO^-^CCR7^+^) and higher frequencies among T terminally differentiated (CD45RO^-^CCR7^-^) (95).

According to the most recent investigations, a distinct pattern of co-inhibitory receptors might also be associated with each species of *Leishmania* involved, such as the case for *L. major* infection, which causes CL. The main role of these checkpoints could be to limit T cell activation during the initial phase of the immunity, during antigen presentation by APCs (96). Specific molecules, e.g. on parasites, might be involved in inducing the expression of checkpoint receptors by different cell subsets upon infection, such as the case of lipophosphoglycan (LPG). This molecule is expressed on the surface of *Leishmania* parasites, and when mice were vaccinated with different LPG concentrations, CD8^+^ T cells upregulated PD-1 on its surface, but not CD4^+^ T cells (97). Additionally, it was recently shown in BALB/c footpad infection with *L. amazonensis* that either anti-PD-1 or anti-PD-L1 treatment reduced the parasite burden and this effect was potentially mediated by CD8^+^IFN-γ^+^ T cells (98). Among the different clinical forms of leishmaniasis, there is evidence supporting the role of PD-L1 as one regulator of anti-VL immunity (85, 99, 100). However, there is very few evidence supporting the same role for PD-L1 during CL. Since both CL and VL take place in different organs in the body, PD-L1 may act distinctly in those sites and its role is not fully understood yet.

It has been shown in *L. donovani* infections that blocking of PD-L1 results in an increased survival of CD8^+^ T cells and partially restores their function, a notion supported by resulting lower parasites numbers upon treatment (101). Other authors have reported the same effect in that infection by *L. donovani* induced PD-1 expression on CD8^+^ T cells and its ligand on splenic DCs (102, 103). Treatment with antibody to block the PD-1/PD-L1 interaction was able to partially restore the function of CD8^+^ T cells, which may indicate that other checkpoint receptors were involved in CD8^+^ T cell exhaustion (102).

Regarding CD4^+^ T cells, Esch et al., 2013 have observed that CD4^+^ T cells from *L. infantum*-infected dogs have a significant increase in PD-1 expression which was associated to some extent with CD4^+^ T cell exhaustion. By blocking PD-L1 *in vitro*, an increase in the production of ROS in monocytes derived phagocytes was found (85). In murine models it has been described that infection with *L. donovani* restricted the expansion of specific CD8^+^ T cells. Moreover, these cells were also more prone to undergo apoptosis and upregulated PD-1, and this dysfunctional phenotype was partially recovered by blocking of PD-L1. Upon PD-L1 inhibition, the authors also detected lower numbers of parasites which might reflect a better infection outcome (102). Furthermore, it has been shown that PD-L1 inhibition restored the function of CD4^+^ and CD8^+^ T cells in the bone marrow of *L. donovani*-infected mice. Upregulation of PD-L1 by macrophages and of PD-1 by CD4^+^, CD8^+^ T and Tregs IL-10^+^Foxp3^+^ upon infection was also observed. Phenotypically, blocking of PD-L1 led to decreased parasite burdens and inhibited autophagy, which is regarded as one of the mechanism used by *Leishmania* to obtain nutrients in the host cell (99). Other studies have also previously described an upregulation of PD-1 and PD-L1 on T cells and macrophages, respectively, upon infection with *L. infantum* or *L. donovani* (85, 102). Nevertheless, it was also demonstrated *Pdl1* ^-/-^ mice infection with *L. major* induced lower frequencies of a population of CD4^+^Ly6C^hi^ effector T cells and higher frequencies of Foxp3^+^ Tregs compared with WT mice (95).

Different species of mammals can act as reservoirs for the parasite, i.e. dogs are an important reservoir for human infection. In the case of dogs VL, it has been shown that there is an impaired CD4^+^ T cell function, IFN-γ production, combined with T cell exhaustion through PD-1 expression (104). On the other hand, one work has shed light upon the role of PD-L1 on regulatory B cells during dog VL, since the specific blocking of PD-L1 on B cells restored Th1 responses (84). The PD-1/PD-L1 axis might exert its function during infection with *Leishmania* by reducing the phagocytic capacity of macrophages. However, it is not yet clear which other cell subsets may also be involved since they can also upregulate PD-L1 upon *Leishmania* infection, e.g. regulatory B cells and DCs. The exact role of PD-L1 expression on these cell subsets is not yet clear, and they might contribute to disease progression and T cell exhaustion. In the case of the PD-1 and PD-L1 pathway it has also been shown for other protozoan infections, blocking the pathway may rescue the function of exhausted cells, such as CD8^+^ T cells and B cells, and this could lead to parasite control and less mortality (105).

PD-L2 is another ligand that binds PD-1 with a two to six fold higher affinity than PD-L1 (106), and it is expressed by macrophages, DCs, bone marrow-derived mast cells, B cells, and intestinal stromal cells (107–111). Expression of PD-L2 is induced by IL-4, IFN-γ and granulocyte-monocyte colony stimulating factor (GM-CSF) (112, 113). More importantly, comparing the roles of PD-L1 and PD-L2 during murine infection with *L. mexicana*, it was observed that *Pdl2* ^-/-^ mice displayed larger lesions and higher parasite burden when compared to *Pdl1* ^-/-^ and WT mice. The most important difference were increased levels of *L. mexicana* IgM and IgG2a that the authors associated with poor disease resolution (114). Those results emphasize a potential role of PD-L2 regulating T-independent (IgM) and -dependent (IgG2a) B cell responses.

In summary, the PD-1 and PD-L1/PD-L2 axis likely regulates immunity against *Leishmania* parasites, and specific blocking of PD-1/PD-L1 potentially boosts T cell responses which are necessary for parasite elimination. Nevertheless, it is not fully clear how this axis works to control parasite proliferation or killing and the role of the different cells that express PD-1/PD-L1.

#### Cytotoxic T Lymphocyte Attenuator 4 (CTLA-4; CD152)

CTLA-4 was discovered many years ago and it is one of the most classical co-inhibitory receptors constitutionally expressed in intracellular vesicles in Foxp3^+^ regulatory T cells (Tregs) and conventional T and B cells during immune activation (115). The first works addressing its function reported that mice lacking CTLA-4 undergo massive lymphocyte proliferation and organ failure (116, 117). CTLA-4 acts by: trans-endocytosis of CD80 and CD86 on APCs to inside the T cells, depleting these ligands (118); inducing IL-10 or TGF-β which down modulate co-stimulatory ligands on APCs (119); finally, it is also suggested that binding of CTLA-4 to CD80/CD86 in DCs induces indoleamine 2,3-dioxygenase (IDO) which degrades tryptophan (120). Lack of tryptophan is also an important mechanism of cell cycle arrest and T cell suppression (121). In 2011, the first anti-CTLA-4 antibody was approved for treatment of melanoma, a type of skin cancer (122, 123).

However, information about the effect of CTLA-4 blocking as an immunotherapeutic intervention against chronic infections is limited. Interestingly, it was observed that susceptible mice treated with anti-CTLA-4 one day post *L. donovani* infection displayed reduced parasite numbers and also induced IFN-γ and IL-4 producing cells (124). However, mice lacking CTLA-4 transducing cytoplasmic tail displayed more susceptibility to infection with *L. major* and increased Th2 responses compared to mice with intact CTLA-4 which were resistant to infection and had stronger Th1 responses (125). Complementary studies have also shown *in vivo* blocking of CTLA-4 promoted Th2 responses and exacerbated disease in *L. major-*infected susceptible mice (126, 127). Additionally, in proliferating CD4^+^ T cells of *Ctla4* ^-/-^ mice increased production of Th2 cytokines was observed (128). Importantly, CTLA-4 engagement results in transforming growth factor (TGF)-β production and this cytokine blocks IFN-γ production inducing parasite survival and proliferation (129, 130). Moreover, blocking of CTLA-4 and neutralization of TGF-β resulted in increased production of IFN-γ and parasite elimination in coculture systems (131, 132). Overall, those remarkable results indicate an important role for CTLA-4 controlling T cell responses during anti-*Leishmania* imunity with important effects mediated by TGF-β.

In humans, expression of CTLA-4 in CD8^+^ T cells of patients infected with *L. panamensis* was described (133). Besides its potential as a potent T cell booster, the role of this receptor during leishmaniasis is still questionable and it needs to be better understood.

#### CD200 (OX-2)

Another axis that has been lately investigated is the engagement of CD200, which is expressed by APCs, with CD200R on T cells (134–136). Following signaling there is formation of a complex of proteins that inhibits Ras activation and results in inhibition of mitogen kinases such as Phosphoinositide 3-kinase (PI3K) and Extracellular Signal-regulated Kinase (Erk) delivering an important inhibitory signal (137–140). Cortez et al., 2011 have shown a correlation between *L. amazonensis* and the expression of CD200 as a mechanism to potentiate its virulence in the host. The authors demonstrated that infection of macrophages with *L. amazonensis*, and not with *L. major*, induces the expression of CD200 transcripts over time (141). Moreover, the lack of CD200 abrogated parasite proliferation in macrophages and this was rescued by the administration of recombinant Fc-CD200. *In vivo*, mice which lack CD200 (*Cd200* ^-/-^) harbored smaller lesions with fewer parasites compared to wild type counterparts. In contrast, the same result was not found in mice infected with *L. major*, indicating that CD200 has an important role at regulating the amount of parasites and the outcome of the infection specifically for *L. amazonensis*. Another interesting point showed by this paper is the improvement of iNOS activity in *Cd200* ^-/-^ mice already 1-h post *L. amazonensis* infection. This suggested that CD200 has a iNOS regulating role during infection. Upon *in vitro* infection of bone marrow macrophages, *Leishmania*-induced increased levels of CD200 in bone marrow macrophages and the lack of CD200 (BMM *Cd200* ^-/-^) inhibited parasite proliferation. Furthermore, it was demonstrated that *Leishmania* (*amazonensis*) DNA recognized by TLR9 is necessary to activate TRIFF and MyD88 and consequently activate CD200/CD200R cis interaction (142). The role of CD200 was also investigated in the context of *L. donovani* infection. The interaction of CD200/CD200R aid activated CD4^+^CD44^+^ T cells to produce IL-4, IL-10, TGF-β, and IL-27. Overall this effect abrogated macrophage effector functions and it was restored by the administration of anti-CD200 which induced more IL-2, IL-12 and IFN-γ by T cells, and therefore activated macrophages to produce NO (143). Additionally, immunization with centrin-deleted *L. donovani* resulted in CD200/CD200R downregulation and consequently suppression of IL-10-producing CD4^+^ T cells and more Th1 cells compared to WT infection (144).

Collectively, the evidence suggests that *Leishmania* can also influence the expression of CD200 as escape mechanism, thus the blocking of this axis holds potential to boost T cell responses and control parasite growth. More investigation is required to address distinct the effect of various parasite species, specific cell types involved, and the relevance for the human situation.

### Stimulatory Checkpoints

#### CD40 and CD40-Ligand (CD40L)

CD40 is a member of the TNF receptor superfamily expressed mainly in B cells and its interaction with CD40L (CD154), expressed on CD4^+^ T cells, stimulates T cells and is crucial to promote germinal center formation and antibody class switching (145–147). It has been shown, *in vitro*, that infection of peritoneal macrophages from BALB/c mice with *L. major* induces different isoforms of CD40-induced N-Ras protein, which in turn induces activation of the ERK-1/2 pathway consequently resulting in less production of IL-12 and more of IL-10 (148). Moreover, the inhibition of CD40 induced N-Ras activation and reduced *L. major* infection. These authors have proposed a model in which the interaction of LPG with TLR2 regulates the different effects mediated by the isoforms of Ras. Thus, this finding highlights one important mechanism by which *Leishmania* species might directly influence immunity through the modulation of CD40-induced Ras protein. Altogether, more investigation is required to broadly clarify those escape mechanisms used by *Leishmania*, and to demonstrate its potential to control *Leishmania* infection *in vivo*.

#### OX40 (CD134)

OX40 is predominantly expressed by activated T cells and when it binds to its ligand OX40L (CD252), expressed by DCs, macrophages and B cells, induces the expression of survival proteins Bcl-2 and Bcl-_XL_ that blocks apoptosis and allows cell cycle progression (149–151). There are evidences indicating OX40/OX40L axis promoting Th2 responses and other refusing this effect (152–155). Zubairi et al. (2004) has shown that treatment of *L. donovani-*infected C57BL/6 mice with OX40-Fc alone or in combination with anti-CTLA-4 diminishes parasite burdens through and IFN-γ and IL-12 dependent manner (155). In contrast, a distinct outcome was observed after infecting *Ox40l* ^-/-^ C57BL/6 mice with *L. donovani* (156). *Ox40l* ^-/-^ C57BL/6 infected mice eliminated parasites in chronic phase and displayed stronger IFN-γ responses compared to WT mice (156). Differently, OX40L-deficient BALB/c mice were resistant to *L. major* infection and exhibited reduced production of Th2 cytokines (154). Likewise, transgenic BALB/c mice overexpressing OX40L displayed pronounced Th2 responses accompanied by a high parasite burden (154). Interestingly, *Ox40l* ^-/-^ BALB/c mice infected with *L. mexicana* or *L. major* developed increased lesions compared to *Ox40l* ^+/+^ counterparts (157). Nevertheless, a Th2 cytokine response bias was verified only for *L. major* infection (157). This pathway has a strong potential to regulate *Leishmania* immunity and it is yet to clarify the exact mechanisms involved during responses against different species.

#### Herpes Virus Entry Mediator (HVEM)

The herpesvirus entry mediator (HVEM) is an important member of the tumor necrosis factor (TNF) superfamily with distinct ligands: lymphotoxin LT-like (LIGHT) and LTα3 that are members of the TNF superfamily; and, B and T lymphocyte attenuator (BTLA; CD272) and CD160 that are members of the Immunoglobulin (Ig) superfamily (158–162). HVEM has cysteine-rich domains 1 and 2 (CRD1, CRD2), where BTLA and CD160 bind to CRD1 and LIGHT binds to CRD2 (163–165). Upon binding, cross linking of HVEM induces a cascade resulting in NF-κB activation (166–168). In another way, HVEM can also act as ligand and pass signals to cells expressing BTLA (169). HVEM has a similar effect (as LIGHT) and controls IL-12 secretion (170). Although there is some evidence on the role of LIGHT/HVEM signaling during *Leishmania* infection (171), the role of one of its main ligands, BTLA, during the infection is not clear yet.

#### Lymphotoxin Exhibits Inducible Expression and Competes With Herpes Simplex Virus Glycoprotein d for Herpes Virus Entry Mediator, a Receptor Expressed by T Cells (LIGHT) or Tumor Necrosis Factor SuperFamily member 14 (TNFSF14)

LIGHT protein is recognized by HVEM and when HVEM is expressed on T cells and interacts with LIGHT from APCs, this results in a T cell stimulatory effect (172). LIGHT can also be expressed by activated T cells, activated NK cells and immature DCs (173, 174). In addition to binding to HVEM, LIGHT can also recognize and bind another receptor known as Lymphotoxin-β Receptor (LTβR) (175). This receptor is found in non-myeloid cells such as epithelial and stromal cells, and myeloid cells such as immature DCs, but not on lymphocytes (176). The axis LIGHT/LTβR is more related with development of the immune system and maintenance of immune responses (177–179). However, interaction of LIGHT with either receptors has been associated with potentiation of inflammatory responses (172). Some data has been presented supporting the role of LIGHT, which is a ligand for HVEM, and an important molecule for the secretion of IL-12p40 by DCs and macrophages (180). LIGHT has an important function in CD8α^+^ DCs during the primary response against *L. major*, but no effect in a secondary response in the presence of CD40 (180). Moreover, LIGHT/HVEM has a critical role controlling parasite burden during the course of *L. donovani* infection through the production of T cell-derived IFN-γ and TNF-α (171). However, LIGHT/LTβR suppress anti-*Leishmania* immunity in the liver of *L. donovani* infected mice (171). **Table 1** summarizes the main role of inhibitory and stimulatory checkpoints in Leishmania infection.

**Table 1:** Role of main immune checkpoints investigated during *Leishmania* infection.

Immune checkpoint		Target cell type	*Leishmania* species or condition	Host organism	Important remarks	Reference
**Inhibitory**	PD-1	CD8^+^ T cells	*L. mexicana*	Humans	CD8^+^ T cells from diffuse form caused by *L. mexicana* have a lower capacity to proliferate, lower IFN-γ production and enhanced PD-1 expression.	(83)
		CD8^+^ T cells	Lipophosphoglycan vaccination	Mice	Vaccination of mice with LPG induces PD-1 expression in CD8^+^ T cells, but not in CD4^+^ T cells.	(97)
		CD8^+^ T cells	*L. donovani*	Mice	PD-1 expressed on CD8^+^ T cells and PD-L1 on DCs. Antibody blocking of PD-1/PD-L1 interaction partially restores CD8^+^ T cell function.	(101, 102)
		CD4^+^ and CD8^+^ T cells	*L. infantum*	Dogs	Increased levels of PD-1 in CD4^+^ and CD8^+^ T cells.	(85)
		CD4^+^ and CD8^+^ T cells	*L. amazonensis*	Mice	Antibody blocking PD-1 or PD-L1 resulted in improved function of CD8^+^ T cells with more production of IFN-γ and reduced parasite burden.	(98)
		CD4^+^ T cells	*L. braziliensis*	Humans	Higher frequencies of CD4^+^PD-1^+^ T cells during active cutaneous leishmaniasis disease compared to post-treated patients and uninfected individuals. Increasing frequencies of PD-1^+^ T cells along with CD4^+^ T cell subset development.	(95)
	CTLA-4 (CD152)	IFN-γ and IL-4 producing cells	*L. donovani*	Mice	Antibody blocking one day post infection reduced parasite burden one month post-infection, and induced IFN-γ and IL-4 producing cells.	(124)
		CD4^+^ and CD8^+^ T cells	*L. major*	Mice	Lack of CTLA-4 transducing cytoplasmic tail increases Th2 response and disease susceptibility when compared to intact CTLA-4 transgenic mice resistant to infection and with strong Th1.	(125)
		CD4^+^ and CD8^+^ T cells	*L. major*	Mice	Blocking of CTLA-4 induces Th2 response and diseases susceptibility.	(126, 127)
		T cells and macrophages	*L. infantum*	Mice	Blocking of CTLA-4 and neutralization of TGF-β induces Th1 cytokines and parasite elimination in coculture systems.	(131, 132)
	PD-L1 and PD-L2	CD8^+^ T cells	*L. donovani*	Mice	Blocking of PD-L1 increases survival of CD8^+^ T cells and reduces parasite burden.	(85)
		CD4^+^ and CD8^+^ T cells	*L. donovani*	Mice	PD-L1 blocking restored CD4^+^ and CD8^+^ T cell function in the bone marrow. Blocking reduced parasite burden and inhibited autophagy.	(85, 102, 124)
		CD4^+^ and CD8^+^ T cells	*L. infantum*	Dogs	*In vitro* blocking of PD-L1 restored CD4^+^ and CD8^+^ T cell function and induced reactive oxygen species in co-culture with monocyte-derived macrophages.	(85)
		B cells	*L. infantum*	Dogs	B cell specific PD-L1 blocking restored Th1 responses through CD3^+^IFN-γ^+^ T cells increase.	(84)
		B cells	*L. mexicana*	Mice	*Pdl2^-/-^* displayed increased lesions and high parasite burden compared to *Pdl1^-/-^* and WT mice. Increased levels of IgM and IgG2a.	(114)
		CD4^+^ T cells	*L. major*	Mice	*Pdl1^-/-^* displayed increased lesions compared to WT mice. Reduced frequency of CD4^+^Ly6C^hi^ effector T cells and higher frequency of Foxp3^+^ Tregs.	(95)
	CD160, 2B4 (CD244), CTLA-4, PD-1, TIM-3	CD8^+^ T cells	*L. panamensis*	Humans	At least 5% of the cells were expressing three or more of those markers.	(133)
	CD200 (OX-2)	Macrophages	*L. amazonensis*	Mice	Infection increases CD200 transcripts. *Cd200* ^-/-^ have smaller lesions and less parasite compared to WT. Effects were not observed upon *L. major* infection.	(141)
		Macrophages	*L. amazonensis*	Mice	Leishmania DNA is recognized by TLR9 and induces CD200-CD200R interaction.	(142)
		CD4^+^CD44^+^ T cells	*L. donovani*	Mice	CD200-CD200R interaction induced IL-4, IL-10, TGF-β and IL-27 by CD4^+^CD44^+^ T cells.	(143)
		CD4^+^ T cells	*L. donovani*	Mice	Centrin-deleted *L. donovani* vaccination resulted in CD200/CD200R downregulation, supression of IL-10, increased Th1 cells (144)	(144)
**Stimulatory**	CD40 and CD40L	Macrophages	*L. major*	Mice	*L. major* infection induces N-Ras through CD40 engagement which results in more IL-10.	(181)
	OX40 (CD134) and OX40L	CD4^+^ and CD8^+^ T cells	*L. donovani*	Mice	Administration of Fc-OX40 diminished parasite burden and induced IFN-γ and IL-12.	(155)
		APCs	*L. major*	Mice	OX40L deficient BALB/c resistant to infection and less production of Th2 cytokines.	(154)
		APCs	*L. major*	Mice	BALB/c overexpressing OX40L presented high parasite burden and strong Th2 responses.	(154)
		APCs	*L. major* *L. mexicana*	Mice	*Ox40l* ^-/-^ BALB/c mice displayed increased lesions compared to WT counterparts.	(157)
	LIGHT (TNFSF14)	CD8α^+^ DCs	*L. major*	Mice	Blockade of LIGHT induces impaired IL-12 and IFN-γ responses.	(170)

### Potential Checkpoints to be Investigated in Leishmaniasis

#### 2B4 (CD244)

2B4 is a member of the signaling lymphocyte activation molecule (SLAM) family and is found on T cells, NK cells, γδ T cells, basophils, monocytes and a subset of memory-phenotype CD8^+^ αβ T cells (182, 183). 2B4 binds CD48 (also known as BCM-1, BLAST-1) in APCs resulting in T cell stimulus (184–187). Egui et al. observed a higher frequency of blood circulating CD8^+^ T cells expressing 2B4 in patients with active leishmaniasis caused by *L. panamensis* (132).

#### T cell Immunoglobulin and Mucin Domain-Containing Protein 3

TIM-3 is another co-inhibitory receptor that was initially identified in IFN-γ producing CD4^+^ and CD8^+^ T cells (188, 189). TIM-3 can also be found in other cell types namely Treg, myeloid cells, NK cells and mast cells (190–193). More recently, TIM-3 has been identified as an important part of a complex of co-inhibitory receptors present in dysfunctional or exhausted T cells (194). Briefly, models indicate that TIM-3 associates with HLA-B-associated transcript 3 (BAT3) to keep cells active through recruitment of tyrosine kinase LCK (195, 196). However, upon binding of either Galectin-9 or CEACAM1 on APCs releases BAT3 and there is recruitment of tyrosine kinase FYN and disruption of immune synapse, phosphatases recruitment and apoptosis (197–199). Therefore, TIM-3 together with other co-inhibitory receptors are potential blocking targets to restore T cell activity (200).

## Limitations of the Available Data

One point to consider is that murine models are important to mechanistically understand the disease and its immunity, however, they cannot mimic all clinical outcomes of human leishmaniasis, such as DCL, MCL, and PKDL (Post-Kala Azar dermal leishmaniasis). Thus, for those clinical forms most of what we know relies on human data. So far, the information on the role of checkpoint molecules during CL is very scarce, and the majority of the work has been done studying human VL.

The role of checkpoint molecules has been extensively described in chronic viral infections in which these receptors are up regulated and, usually, suppress the activity of CD8^+^ T cells. Their capacity to suppress other cell subsets has been correlated with the co-expression of different checkpoint molecules and the production of key cytokines such as IL-10 (201). However, the role of CD8^+^ T cells during *Leishmania* infections is still not clear yet and depending on the level of its activity, CD8^+^ T cells might be involved with the production of local IFN-γ and activation of macrophages to kill parasites or might be involved with immunopathology and worse tissue outcome.

On the other hand, the role of CD4^+^ T helper 1 (Th1) cells has been better described due to its multifunctional production of cytokines, such as IFN-γ and TNF-α, and, consequently, activation of macrophages to kill parasites. However, the role of checkpoint molecules being expressed on CD4^+^ T cells is not clear yet, whether these molecules indicate an activated cell phenotype or depending on the degree of co-expression an exhausted cell (94, 202–206). So far, data suggests that during CL caused by distinct species of *Leishmania* there is an important upregulation of immune checkpoints in CD4^+^ and, especially, CD8^+^ T cells. However, the role those pathways play during infection is still inconclusive and the parasites might use some of them to bypass immunity.

Leishmaniasis imposes a complex scenario in which different species of one parasite united by the same genus can cause a broad spectrum of clinical manifestations and thus different immune responses. Checkpoint molecules appear to have a distinct role for immunity elicited by the different species of *Leishmania* or the different clinical outcomes.

## Translational Implications

One central question to ask is how much of this knowledge can be translated to human leishmaniasis? Therapies using anti-PD-1, anti-PD-L1, and anti-CTLA-4 blocking antibodies were approved by the United States Food and Drug Administration (FDA) and became an important treatment for different types of cancer (207–212). Even though there is evidence that in VL the inhibition of some of those co-inhibitory receptors can restore T cell function and control parasite burden, no clinical studies have been performed.

Lack of translational implications brings two facts for reflection: recombinant antibody administrations are still expensive and therefore far from the governments and the main populations affected by leishmaniasis; and there are not enough scientists and funding to sponsor those clinical studies. Biological treatments are still costly and restricted to populations who can afford that (213, 214). It is still unclear whether these treatments are going to expand and consequently drop in price so that treatment of other diseases may benefit as well. Although *Leishmania* infections cause disease in one million individuals every year, the research in the field is still far beyond necessary for the magnitude and complexity of the problem. In this scenario, it is difficult to foresee translational studies in leishmaniasis patients needed in the upcoming future. As a result of global warming and population growth, substantial funding is need for research in leishmaniasis.

## Concluding Remarks

**Figure 3:**
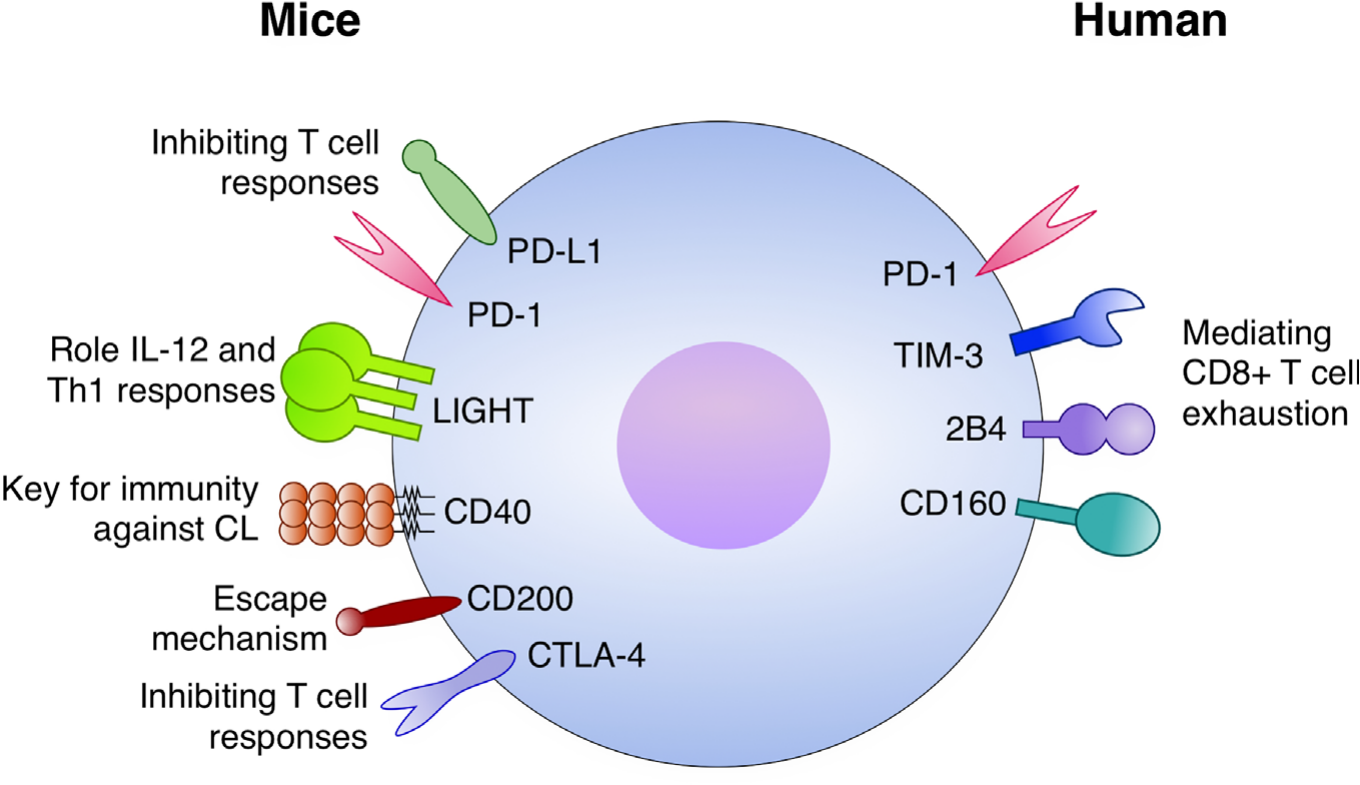
Immune checkpoints with a role in Leishmaniasis. In mice models: The PD-1/PD-L1 axis inhibits T cell responses, both CD4^+^ and CD8^+^, during infection. LIGHT has an important role for IL-12 and Th1 responses. CD40 is a key factor for immunity against cutaneous leishmaniasis (CL). CD200 expression is implicated with escape mechanisms by the parasite and CTLA-4 is an important inhibitor of T cell responses against *Leismania*. In humans, PD-1 TIM-3, 2B4, and CD160 have been potentially mediating CD8^+^ T cell exhaustion.

Immune checkpoints either stimulatory or inhibitory are induced during the development of immunity against *Leishmania* (**Figure 3**). Stronger data for VL indicates that both PD-1 and PD-L1 are upregulated by CD4^+^ and CD8^+^ T cells. Moreover, in this setting the blocking strategy under the course of infection specially restored CD8^+^ T cell function and promoted a stronger Type I responses with IFN-γ activating macrophages and reducing parasite burden. The mechanisms behind the function of immune checkpoints during dermatotropic *Leishmania* infections is even more unclear, but data suggests that LIGHT and CD40 have important roles in maintaining immunity against *Leishmania species* inducing CL. The most promising data obtained with *L. major* suggested that LIGHT blockade impairs IL-12 secretion and Type I responses. Now it will be important to deeply characterize those pathways dissecting important questions: which are the cell types participating in this regulatory process and how those mechanisms act at the molecular, cellular and tissue levels. Translational application of those studies are still far from the field; however, they are shedding light on important mechanisms of the human system to control intracellular pathogens such as *Leishmania*.

## Author Contributions

RS wrote the paper with input from ES. All authors contributed to the article and approved the submitted version.

## Funding

This study was supported by the Universal-CNPq (Processo: 424889/2018-8) to RS and SFB 1292 (Deutsche Forschungsgemeinschaf, DFG) and the Center for Molecular Medicine Cologne (CMMC) to ES.

## Conflict of Interest

The authors declare that the research was conducted in the absence of any commercial or financial relationships that could be construed as a potential conflict of interest.
